# Fatty Acids of Ten Commonly Consumed Pulses

**DOI:** 10.3390/molecules27217260

**Published:** 2022-10-26

**Authors:** William Craig Byrdwell, Robert J. Goldschmidt

**Affiliations:** Methods and Application of Food Composition Laboratory, Agricultural Research Service, U.S. Department of Agriculture, Beltsville, MD 20705, USA

**Keywords:** legumes, beans, peas, lentils, GC-FID, GC-MS, oxidation products

## Abstract

Gas chromatography (GC) with flame ionization detection (FID) and mass spectrometry (MS) detection were used to characterize the fatty acid (FA) compositions of ten commonly consumed (i.e., market class) pulses. Lipids from ground pulses were extracted using a classical chloroform/methanol extraction and quantified by GC-FID with structural confirmation by GC-MS. Principal component analysis (PCA) was applied to FA compositions of the pulse extracts, and the pulses clustered into three distinct groups: one rich in linolenic acid, 18:3 (carbon number:unsaturation, C:U), one rich in 16:0, and one in which 18:1 was highest, along with predominant 18:2. These ten pulses averaged 46.1% linoleic acid (18:2), 22.7% oleic acid (18:1), 18.0% palmitic acid (16:0), and 7.6% linolenic acid (18:3). Individual values ranged widely, with 18:2 ranging from 26.0% in black beans to 48.4% in garbanzo beans. The greatest difference was in 18:3, which ranged from 2.2% in garbanzo beans to 38.8% in pinto beans. Oxo-FA were observed in all ten samples, and the distribution of oxo-FA in the samples also varied. Overall, the very different FA compositions of pulses lead to the possibility of breeding and genetic modification between pulses to produce the most desirable FA composition for nutritional benefit.

## 1. Introduction

Legumes are plants of the family *Fabaceae*, also called *Leguminosae*, which bear seeds in pods, and the seeds are known as pulses. These include beans, peas, lentils, and chickpeas. The United Nations Food and Agriculture Organization has limited the definition of pulses to include only “crops harvested solely for dry grain, thereby excluding crops harvested green for food, which are classified as vegetable crops, as well as those crops used mainly for oil extraction and leguminous crops that are used exclusively for sowing purposes” [1].

Crops used mainly for oil extraction, or oilseeds, such as soybeans and canola (low erucic acid rapeseed) have been studied extensively for decades, since they have long been nutritionally important commodities. The fatty acid compositions of oilseeds were determined using fractional crystallization prior to the wide use of gas chromatography [2]. As almost every new analytical method was developed (GC, high performance liquid chromatography (HPLC), mass spectrometry (MS), etc.) it has been applied to oilseed analysis, due to ongoing interest in more detailed and accurate FA and other lipid compositions.

Gas chromatography is now a rather mature technique, with instruments and columns having evolved over the decades to include the modern era of higher resolution capillary columns that provide better separations than ever allowed on classical GC columns. Nowadays, the use of capillary columns allows the separation of complex mixtures containing double bond isomers, branched-chain isomers, and more.

Pulses, in contrast to oilseeds, are low in oil, typically around 2% to 3% of seed mass, though some species, such as chickpeas (*Cicer arietinum*) may have 6% or more fat content [3,4,5,6,7,8]. The lipid contents of pulses have received much less attention than those of oilseeds. Nevertheless, as pulses are nutritionally important globally, their lipid contents are of interest [3]. The long development of improved GC instruments and columns can now be leveraged to apply the latest, most high-resolution GC techniques to identify and quantify the fatty acids in pulses with great specificity and detail regarding FA isomers.

The fat content of oilseeds consists primarily of triacylglycerols (TAG) which are stored in the seed oil bodies [9]. Pulses and other legume seeds, although they have lipid particles analogous to oil bodies for storing TAG, tend to store more protein and carbohydrate [10,11]. Phospholipids (PL) have been reported to contribute 30% to 60% of the lipid content of some pulses, with TAG making up the bulk of the remainder [12,13,14]. Chickpeas were reported to have about 20% PL and 60% TAG [15]. Froese et al. [10] found for wax beans (*Phaseolus vulgaris*) that smaller, high density lipid particles (HDP) had about 40% each of TAG and PL, but that larger, low density lipid particles (LDP) had near 80% TAG. Yoshida et al. found for kidney beans [13] and for peas [12] that there were relatively higher levels of 18:3 and lower levels of 16:0 in TAG as compared to PL, though the percentages of the other FA were similar in the two fractions.

In this report, we describe the FA compositions of ten commonly consumed market class pulses, determined using GC-FID and GC-MS on a long GC column using a method that was optimized for the complex isomer mixtures of milk extracts. The FA compositions of pulses are simpler than milk, and the column provided outstanding separation of pulse extract FAs, allowing isomers to be identified and quantified.

Principal component analysis (PCA) and hierarchical cluster analysis (HAC) were applied to the FA compositions determined for the 10 pulse lipid extracts, for comparison to similar analyses done for the TAG content of these samples (manuscript submitted), and, in the case of HCA, done for their soluble sugar content [16].

In addition, we report on some oxygenated FA that were observed by GC-FID and GC-MS and observed in TAG by LC-MS (manuscript submitted). Oxygenation may have occurred during sample storage; however, the characterization and appearance of the oxygenated FA in the chromatograms may be of interest to others investigating FA of pulses. The detailed FA compositions of the ten pulse samples, including isomers and the oxygenated FA, determined by GC-FID and confirmed by GC-MS are presented here.

## 2. Results and Discussion

Lipid residue yields from extractions using the method of Folch et al. [17], with one exception, ranged between 1.9% (butter beans and baby lima beans) and 2.6% (black and navy beans) of ground sample mass. Garbanzo bean yields were higher, at 6.2%. These estimates are within the ranges reported elsewhere for similar pulse samples [3,6,12,15,18,19,20,21], though estimates for a given genus and species can be significantly lower or higher. Sample differences accounted for much of the variation in estimates, but the solvent systems used for extraction can also have significant effects. Caprioli et al. [6] found that Folch-type extractions (chloroform-methanol) were the most efficient of several they tested of commonly used solvent systems for total lipid extraction from pulses and that pre-hydrating ground samples increased the amount of lipid extracted by as much as 30% over Folch extractions with no pre-hydration.

Table 1 gives the FA mole percentage compositions of the pulse samples as determined by GC-FAME analysis. Relative quantitation was determined from FID chromatograms. The table includes only FAME peaks that were present at ≥0.01% and with identification supported by PCI-CH_4_ MS and RT matching. Grouped by carbon number and degree of unsaturation (C:U), 22 different FA were identified in the samples, and 31 when isomers are counted separately.

Typical of plant sources, four of the FA groups were by far the most abundant for these samples: 16:0, 18:1, 18:2, and 18:3. Combined, they contributed between 91% (butter beans) and 96% (pinto, navy, and garbanzo beans) of the total FA contents. When the next most abundant species, 18:0, is included, the percentages increased to between 95% and 98% of the total FA contents. For only one sample did a FA other than these five contribute as much as 1% of the total FA content: 22:0 and 24:0 in black-eyed peas.

The several 16:1 isomers collectively contributed from 0.4% (black-eyed peas) to 2.8% (garbanzo beans) of the total 16-carbon FA content. 18-carbon FA are predominantly unsaturated. Stearic acid, 18:0, contributed from 1.7% (garbanzo beans) to 8.8% (butter beans) of the total 18-carbon FA content. 18:1 isomers contributed from 7.9% (black-eyed peas) to 39.8% (garbanzo beans), 18:2 isomers from 33.4% (black beans) to 65.1% (baby lima beans), and 18:3 isomers from 2.6% (garbanzo beans) to 50.6% (pinto beans) of the total 18-carbon FA content.

**Figure 1:**
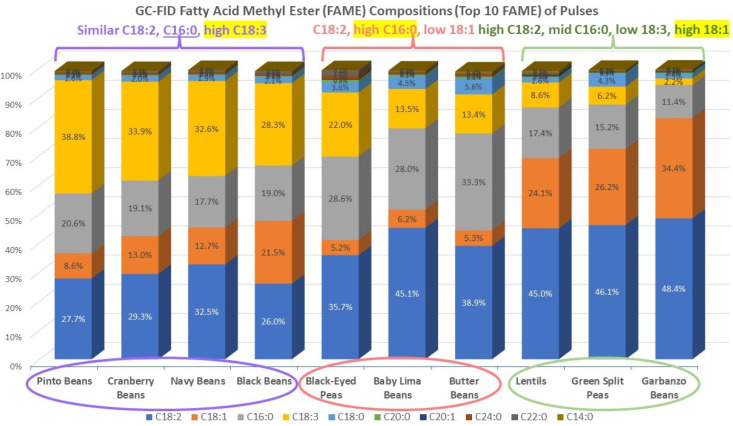
Stacked bar chart showing the percentages of the ten most abundant FA, on average, of the ten pulse samples. The samples are arranged in order of decreasing percentage of 18:3, from left to right. Colored ovals correspond to groups in Figure 2.

Figure 1 is a stacked bar chart of the 10 most populous FA, on average, found for the pulse samples. The samples are arranged according to decreasing relative abundance of 18:3, from left to right. The stacked columns illustrate the dominance of the four most populous FA and suggest that the samples fall into three groups based on the relative percentages of these FA. Group 1 is comprised of the four Phaseolus vulgaris samples, with the most obvious shared feature being the relatively high percentage of 18:3. Group 2 contains the two Phaseolus lunatus samples plus the Vigna unguiculata sample. The most obvious distinguishing feature of these samples is that they are relatively high in 16:0. Group 3 contains three samples none of which are from the same genus. These samples were relatively lowest in 18:3 and highest in 18:1.

**Figure 2:**
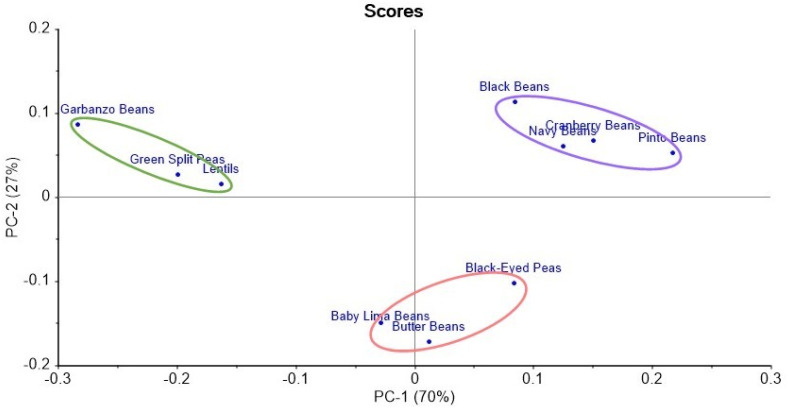
Scores plot from Principal Component Analysis of the complete FA compositions by C:U of the ten pulse samples. Ovals added to highlight clusters.

Principal component analysis (PCA) using the complete compositions of FA (C:U) separated the samples into the three groups suggested by Figure 1. The scores plot in Figure 2 shows that the first principal component (PC-1), which accounted for 70% of the total variance, was responsible for most of the differences between Groups 1 and 3.

The second principal component (PC-2), which accounted for another 27% of the total variance, represented most of the differences between Group 2 and the other two groups.

**Figure 3:**
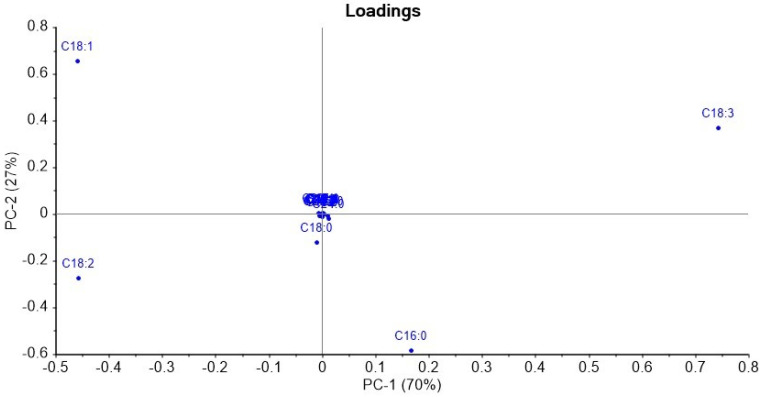
Loadings plot from Principal Component Analysis of the complete FA compositions by C:U (carbon number: unsaturation) of the ten pulse samples.

The loadings plot in Figure 3 indicated that the 18:3 FA relative percentage strongly influenced PC-1 and was positively correlated with it. Because of this, 18:3 was chosen as the basis for sorting in Figure 1. The 18:1 and 18:2 relative percentages also contributed to PC-1, but they were negatively correlated with it. The 16:0 relative percentage had the weakest influence on PC-1 of the four major FA and was positively correlated with it.

The 18:1 and 18:3 FA relative percentages were positively correlated with PC-2, and the 18:2 and 16:0 FA relative percentages were negatively correlated with it. 18:0 had a small influence on PC-2 and less on PC-1, and the other FA contributed very little to either of the first two principal components.

**Figure 4:**
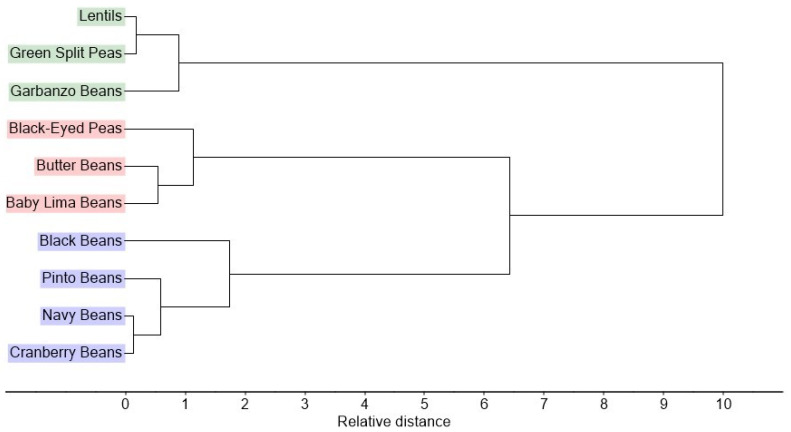
Hierarchical Cluster Analysis dendrogram showing clusters based on the complete FA compositions by C:U of the ten pulse samples. Colors match ovals in Figure 2.

Hierarchical Cluster Analysis (HCA) was also applied to the relative percentages of the complete set of FA (by C:U) found for the pulse samples. Figure 4 shows that HCA grouped the samples into three distinctive clusters having the same members as the three groups determined by PCA.

The ten samples analyzed in this work were also analyzed by ultra high performance liquid chromatography (UHPLC) with MS detection for TAG composition, reported elsewhere (manuscript submitted). PCA and HCA were also applied to the TAG results, and the resultant three sample groups were the same as the groups described above. These ten samples are a subset of 23 pulse samples previously analyzed for soluble sugar content [16]. HCA applied in that work separated the 23 samples into five clusters. There are similarities with the present work in how the subset of 10 samples were grouped, but also some differences. For example, baby lima beans and black-eyed peas were grouped together in both works, as were pinto, navy, and black beans. However, cranberry beans were included in the latter group by HCA of FA content but put in a different group by HCA of soluble sugars content. The latter group included green split peas, but did not include lentils or garbanzo beans, which were in two other, separate groups.

FA compositions presented here are similar to other published reports for pulse samples of the same genus and species, yet in some cases published reports are significantly different. For example, the FA percentages reported here for the chickpea sample are within the ranges found for Canadian chickpeas of the Kabuli variety as reported in a review by Jukanti et al. [5]. In the same review, percentages of 18:2 are given as about 20% higher and percentages of 18:1 about 30% lower for Canadian chickpeas of the Desi variety as compared to the Kabuli variety. Grela and Gunter [20] reported percentages similar to ours for the four major FA found for a Lens culinaris sample, but for a Pisum sativum sample, they reported close to equal amounts of 16:0 and 18:1, and for a Phaseolus vulgaris sample, they reported the 18:2 percentage at nearly 3.5 times the percentage of 18:3. Genetic differences among varieties and cultivars of the same genus and species can account for differences in FA composition, as can growing conditions [22].

Peak areas from the FID chromatograms attributable to the FA of the extraction internal standard (EIS) containing 9:0, 13:0, 17:0, and 21:0 were excluded from the relative quantitation analysis and are not included in Table 1 or in the PCA and HCA analyses. The sum of the EIS component areas, including any endogenous contributions, was 10% or less of the total integrated FID peak areas for each sample. Additional analyses without additions of the EIS were made for black, navy, and pinto beans, and small amounts of 17:0 and 21:0 were found, but 9:0 and 13:0 were not observed. The sums of the endogenous 17:0 and 21:0 peak areas were estimated to be less than 0.5% of the total integrated peak areas for black, navy, and pinto beans. Although there was no direct determination of endogenous 9:0, 13:0. 17:0, and 21:0 for the other seven samples, peak areas for those FA in the samples to which EIS was added were similar to those found for black, navy, and pinto beans, and, if present, endogenous levels of EIS FA are presumed to be similarly low.

For each sample, several other peaks were observed in the FID chromatograms that were not attributable to the FA listed in Table 1. Some of these were possible additional 18:1, 18:3, and 20:1 isomers, as suggested by RT, but they were excluded because their FID signal levels were <0.01% of the total integrated peak areas and because of a lack of supporting PCI-CH_4_-MS evidence. Squalene, with RT just before that of the 23:0 FAME, was observed in all samples except for lentils. Squalene is composed of carbon and hydrogen only and was not affected by the basic FAME treatment used. It yielded an [M + H]^+^ ion under PCI-CH_4_ conditions and was identified by MS and RT comparison to a standard. Several TAGs containing oxygenated FA were observed by LC-MS analysis (manuscript submitted), and the presence of a total of five different oxygenated FA were confirmed by PCI-CH_4_ GC-FAME analysis. A suspected sixth oxygenated FA was also observed. Although the PCI-CH_4_ peak mass spectra did not precisely identify the structures of the oxygenated FA, they were sufficient for establishing FAME molecular masses. The molecular masses were consistent with mono-, di-, and tri-enoic 18-carbon FAME with the substitution of a hydroxyl group for a hydrogen or insertion of an oxygen atom across a site of unsaturation to form an epoxide. Thus [M + H]^+^ ions of 18:1, 18:2, and 18:3 FAME were observed at *m*/*z* 297, 295, and 293, respectively, but [M + H]^+^ ions of the corresponding oxygenated FAME were observed at *m*/*z* 313, 311, and 309.

**Figure 5:**
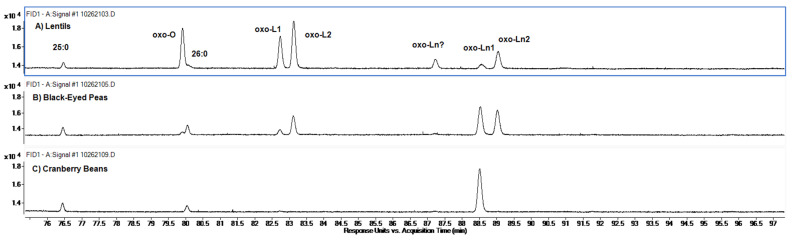
Partial FID chromatograms showing peaks for oxygenated FAME observed for (**A**) lentils, (**B**) black-eyed peas, and (**C**) cranberry beans. The peak labeled oxo-O corresponds to an oxygenated 18:1 FAME. The peaks labeled oxo-L1 and oxo-L2 correspond to oxygenated 18:2 FAME. The peaks labeled oxo-Ln1 and oxo-Ln2 correspond to oxygenated 18:3 FAME. The peak labeled oxo-Ln? could not be characterized by the PCI-CH_4_ MS data, but it is suspected to be a third oxo-Ln isomer.

Partial FID chromatograms including the peaks for oxygenated FAME observed for lentils, black-eyed peas, and cranberry beans are shown in Figure 5. The oxygenated FAME eluted toward the end of the chromatographic runs, well separated from the non-oxygenated 18:1, 18:2, and 18:3 FAME peaks. An oxygenated FAME corresponding to 18:1, labeled as oxo-O in Figure 1, had a RT just before that of the FAME of 26:0, the latest eluting of the FAME listed in Table 1. Two oxygenated FAME corresponding to 18:2, labeled oxo-L1 and oxo-L2, eluted close together a couple of minutes after oxo-O, and two oxygenated FAME corresponding to 18:3, labeled oxo-Ln1 and oxo-Ln2, eluted close together several minutes later. A sixth late eluting peak, eluted between the oxo-L and oxo-Ln peaks, but closer to the latter, was also observed and can be seen in Figure 1. The PCI-CH_4_ signal from this peak was not strong enough to confirm its identity as an oxygenated FAME, but from its position in the chromatograms, it may be a third oxo-Ln isomer. Observation of a single oxo-O isomer, two oxo-L isomers, and three oxo-Ln isomers would be consistent with the numbers of isomers expected from epoxide formation at the carbon-carbon double bond sites of O, L, and Ln, by far the most abundant 18:1, 18:2, and 18:3 isomers, respectively, in the samples.

Several other small peaks were observed in the chromatographic region shown in Figure 5, but they were too small to be seen in the figure and did not give useful PCI-CH_4_ MS data.

Table 2 gives the mole percentages of squalene, total oxo-FA, and total non-oxo-FA as determined from GC-FID peak areas for each of the ten samples. The mole percentages were determined in the same way used for the FA compositions in Table 1, but with the inclusion of squalene and the oxo-FA. Squalene relative amounts ranged from just under 0.005% (garbanzo beans) to 0.7% (navy beans). The relative amounts of oxo-FA ranged from 0.09% in butter beans to 3.3% in lentils.

The 18-carbon oxo-FA distributions did not closely follow the non-oxygenated, unsaturated 18-carbon FA distributions (18:1, 18:2, and 18:3), as shown in Table 3. For example, oxo-Ln isomers accounted for over two thirds of the total amount of oxo-FA for black-eyed peas, whereas 18:3 FA isomers comprised about one third of the total non-oxygenated, unsaturated 18-carbon FA amount. For cranberry beans, oxo-Ln accounted for almost all the total oxo-FA content, with a small amount of oxo-L and no observed oxo-O, but the non-oxygenated, unsaturated 18-carbon FA were more evenly distributed, with a little less non-oxygenated 18:2 than 18:3, and 18:1 at less than half the 18:2 amount.

For all samples, the oxo-O relative amounts were smaller and the oxo-Ln relative amounts were larger than the corresponding relative amounts of non-oxygenated 18:1 and 18:3 FA, respectively. The oxo-FA compositions, relative to the corresponding non-oxygenated, unsaturated 18-carbon FA compositions, were consistent with increasing susceptibility to oxidation with increasing unsaturation [23,24,25]. Thus one might suspect that the oxo-FA contents were due to oxidation occurring during long-term sample storage (or handling), although Table 2 shows that the degree of oxidation among the samples varied.

A more detailed look at the oxo-FA distributions is given in Table 4, where the oxo-L and oxo-Ln components are expanded to show the isomer contributions. The only obvious grouping suggested by the distributions was that of the four Phaseolus vulgaris samples, which were found to contain the oxo-Ln1 isomer exclusively, or nearly so. The distributions for baby lima beans and butter beans were also dominated by the oxo-Ln1 isomer, though they also contained measurable amounts of both oxo-L isomers. Lentils contained higher levels of oxo-Ln2 than oxo-Ln1. The levels of oxo-Ln1 and oxo-Ln2 were about equal in garbanzo beans, and that was also the case for black-eyed peas.

For the samples in which oxo-L was found, there was also variation in which isomer was more prevalent. Oxo-L1 was present at higher levels than oxo-L2 in green split peas and in baby lima beans, for example, but the reverse was true for lentils, garbanzo beans, and black-eyed peas.

Although enzymatic involvement could account for why there was such variation in the oxo-FA distributions among the samples if oxidation were happening primarily during storage and handling [26], it could also be indicative of metabolic formation of oxidized FA, as is known to occur in some plants [27].

## 3. Materials and Methods

### 3.1. Samples

Ten ground pulse samples were provided by Dr. Raghavendhar R. Kotha [16], received from Prof. John Finley at Louisiana State University. Samples were stored at −80 °C and brought to room temperature shortly before weighing and extraction. Samples grouped according to genus and species were as follows: four *Phaseolus vulgaris* (black, cranberry, navy, and pinto beans), two *Phaseolus lunatus* (baby lima and butter beans), and one each of *Vigna unguiculata* (black-eyed peas), *Cicer arietinum* (garbanzo beans), *Pisum sativum* (green split peas), and *Lens culinaris* (lentils).

### 3.2. Reagents and Solvents

Optima Grade Methanol was from Fisher Scientific (Fair Lawn, NJ, USA). Chloroform, heptane, and isooctane were HPLC grade from Sigma-Aldrich (St. Louis, MO, USA). Water was from an in-house Millipore (Bedford, MA, USA) Milli-Q Water Purification System. Potassium hydroxide (Aldrich Chemical Company, Milwaukee, WI, USA) and potassium chloride (Fisher Scientific) were reagent grade.

### 3.3. Extraction of Lipids from Pulse Samples

Lipid extractions were based on the method of Folch et al. [17]. Ground pulse samples (2.2 g to 2.5 g) were placed in 50 mL glass screw top tubes with Teflon-lined caps. To each sample, 20 mL Folch solvent (2:1 chloroform:methanol) and 5 mL of an extraction internal standard were added. The EIS was for use in concurrent LC-MS analysis and contained 0.02062 µmol/mL each of tri-9:0, tri-13:0, tri-17:0, and tri-21:0 TAGs, plus 2.0 µg/mL of vitamin D_2_ in Folch solvent. Samples were magnetically stirred, with periodic vortexing, for 30 min. They were then centrifuged at 1000× *g* rpm for two minutes, and the supernatants were transferred by Pasteur pipet to a second set of 50 mL glass screw top tubes. The supernatant was washed with a 0.2 × volume of 0.9% KCl. Phase separation was again aided by centrifuging, and the lower, chloroform layer was collected. The extraction and washing procedures were then repeated on the samples using 20 mL portions of Folch solvent. For each sample, the two washed chloroform portions were combined, and a 5 mL portion was taken for GC analysis, with the remainder reserved for LC-MS analysis (reported elsewhere (manuscript submitted). For the GC portion, solvent evaporation to constant mass was performed at room temperature under a stream of nitrogen gas. Residue masses for GC analysis were 8 to 10 mg except for chickpeas, for which the residue mass was 30 mg. Residues were dissolved in 1.5 mL isooctane and treated for FAME derivatization.

### 3.4. FAME Derivatization

A 0.25 mL portion of 2 M KOH in methanol was added to each of the residues dissolved in isooctane. Samples were vortexed for 30 s and kept at room temperature for 30 min. The top, isooctane layers were then transferred by Pasteur pipet to GC sample vials for analysis by GC-FID and GC-MSD.

### 3.5. GC Standards

Multi-component FAME standards GLC 68B (16 FAME from C14 to C24) and GLC 463 (52 FAME from C4 to C24) were obtained from Nu-Check Prep (Elysian, MN, USA). Methyl heneicosanoate (21:0) and Methyl tricosanoate (23:0) were also obtained from Nu-Check Prep. Methyl pentacosanoate (25:0) was obtained from Santa Cruz Biotechnology, Inc. (Dallas, TX, USA). Methyl hexacosanoate (26:0) was obtained from Matreya LLC (Pleasant Gap, PA, USA). Standard L6031, containing the FAME of the eight geometrical isomers of 9, 12, 15-18:3, and squalene were obtained from Sigma-Aldrich. An isomerized corn oil sample, previously described [28] and derivatized to produce FAME as above, was used to identify the geometrical isomers of 9, 12-18:2. Excluding the one derived from corn oil, FAME standards were prepared as isooctane or heptane solutions at total FAME concentrations ranging from 0.2 mg/mL to 2 mg/mL.

### 3.6. Gas Chromatography and Mass Spectrometry

Two GC systems were used. One system was equipped with a single FID detector, and one with the effluent split between a mass selective detector (MSD) and an FID.

#### 3.6.1. GC and GC-MS Apparatus

(a) Gas Chromatograph—Agilent Technologies (Santa Clara, CA, USA) 6890N GC with G2613A injector, split/splitless inlet, flame ionization detector (FID), and using OpenLab CDS ChemStation Edition for GC Systems (Rev. C.01.05 [Build 35]) software.
(b) GC-MS—Agilent Technologies 5975C Inert XL EI/CI MSD with 7890A GC System and G4513A injector, split/splitless inlet, G3180B two-way splitter with makeup gas, FID, and using MassHunter GC/MS Acquisition (B.07.00 SP1.1549) software.

#### 3.6.2. 6890. N GC-FID Method

(a) GC Column—Supelco (Bellefonte, PA, USA) SP-2560 (100 m × 0.25 mm × 0.2 µm film thickness).
(b) Carrier Gas—Hydrogen with operation in constant flow mode at 1.3 mL/min.
(c) Inlet—Temperature 250 °C; 1 µL injection; operation in split mode at 100:1 split.
(d) FID—Temperature 260 °C with total hydrogen flow at 35 mL/min, air flow at 350 mL/min, and make-up gas (N2) flow at 25 mL/min.
(e) Oven—Initial temperature 70 °C, temperature gradient of 7.8 °C/min to 130 °C; gradient of 3.1 °C/min to 189 °C with 2 min hold; gradient of 1.6 °C/min to 200 °C; gradient of 3.2 °C/min to 230 °C with 22 min hold.
(f) Run Time—67 min.

#### 3.6.3. 5975. C/7890A GC-MSD-FID Method

(a) GC Column—Supelco SP-2560 (100 m × 0.25 mm × 0.2 µm film thickness).
(b) Carrier Gas—Helium with operation in constant flow mode at 1 mL/min.
(c) Inlet—Temperature 250 °C; 1 µL injection; operation in split mode at 50:1 split.
(d) FID—Temperature 260 °C with hydrogen flow at 35 mL/min, air flow at 350 mL/min, and make-up gas (N_2_) flow at 25 mL/min.
(e) Oven—Initial temperature 70 °C, temperature gradient of 5 °C/min to 130 °C; gradient of 2 °C/min to 180 °C; gradient of 1 °C/min to 196 °C; gradient of 2 °C/min to 230 °C with 30 min hold.
(f) Run Time—100 min.
(g) MSD Parameters—Operation in positive chemical ionization mode with methane reagent gas (PCI-CH_4_); scan from *m*/*z* 50 to *m*/*z* 450 at 1.9 Hz.

### 3.7. Calculations

FID peaks were manually integrated within the Agilent ChemStation data analysis software, and retention time (RT) versus peak area data were transferred to an Excel spreadsheet for further analysis. FAME mass percentages were obtained directly, and these were converted to mole percentages. Molar response factors based on the FID responses of the FAME of the GLC 463 standard were applied to the mole percentages. The corrected molar percentages were used for construction of Table 1 above. Principal component analysis (PCA) and hierarchical cluster analysis (HCA) were performed using Unscrambler X (formerly Camo Analytics, now AspenTech, Bedford, MA, USA).

## 4. Conclusions

The FA compositions of pulses were less complex than those of samples such as dairy products and meats, so the use of highly polar 100 m capillary columns was highly beneficial for isomer separation of pulse FA. Five 16:1 isomers and three isomers each of 18:1, 18:2, and 18:3 were included in the compositions reported here, but it is suspected that several additional isomers were present at levels too low for confirmation by PCI-CH_4_ MS. Oxo-FA isomers present in these samples were also separated.

The FA compositions presented here, determined using basic (KOH) FAME derivatization, included the contributions from TAG and PL, as well as small amounts of other lipid components, though not free FA, which are presumed to be minor. Reports on kidney beans [13] and peas [12] showed that although their FA compositions were similar for most FA, they differed in the relative amounts of 16:0 and 18:3. In future work, we will add a class separation to examine the TAG and PL FA compositions of pulse samples separately.

PCA was helpful in identifying trends and grouping the ten pulse samples into clusters with related FA compositions. HCA produced the same clusters. Since FA and TAG compositions are correlated, it is perhaps not surprising that the sample clusters from PCA and HCA reported here are the same as those found for the TAG compositions of these samples (manuscript submitted), though any discrepancies as might arise from a different or larger dataset could be informative.

The differences in the oxo-FA compositions found for these pulse samples probably reflect enzymatic or metabolic differences. The four *Phaseolus vulgaris* compositions were all dominated by the oxo-Ln1 isomer. The two *Phaseolus lunatus* samples are less similar, which may be indicative of age at harvest.

*Future Work.* While the pulses examined here are commonly available ‘market class’ pulses, there are other genotypes that are being bred for a variety of purposes, including drought resistance. We will investigate how breeding for some traits affects the FA compositions. We will extend this work to a series of breeding lines of *Phaseolus vulgaris* to identify cultivars with fatty acid compositions substantially different from the market class samples. We will also analyze cooked pulses to be used in dietary intervention studies, to provide FA compositions of dietary components actually consumed.

## Figures and Tables

**Table 1:** Fatty acid compositions of pulses determined as the fatty acid methyl esters (FAME) by gas chromatography with flame ionization detection. Left-justified values are fatty acids by carbon number:degree of unsaturation. Right-justified values are the compositions of isomers of the value above. Fatty acids and most abundant FA percentages in bold.

FA	Baby Lima Beans	Black Beans	Black-Eyed Peas	Butter Beans	Cran-berry Beans	Gar-banzo Beans	Green Split Peas	Lentils	Navy Beans	Pinto Beans
**C12:0 ^a^**	0.00	0.00	0.00	0.00	0.00	0.01	0.00	0.03	0.00	0.00
**C14:0**	0.20	0.12	0.15	0.44	0.07	0.15	0.25	0.52	0.06	0.10
**C15:0**	0.42	0.22	0.08	0.55	0.13	0.09	0.24	0.19	0.12	0.21
**C16:0**	27.95	18.99	28.62	33.26	19.12	11.35	15.17	17.37	17.70	20.59
**C16:1**	0.18	0.37	0.12	0.24	0.34	0.33	0.17	0.20	0.20	0.35
^b^ Δ9*c*-16:1 (Po)	50.92	65.37	17.68	42.74	64.84	78.25	44.78	50.91	62.62	66.65
Δ9*t*-16:1 (Po)			53.72							
^c^ 16:1X1		6.40		11.05	7.65	3.70	25.35	12.67		7.08
16:1X2	23.31	16.49	28.60	20.84	14.36	12.45	29.87	36.42	21.69	10.54
16:1X3	25.78	11.73		25.37	13.15	5.60			15.69	15.73
**C17:1**	0.03	0.12	0.02	0.04	0.19	0.01	0.03	0.09	0.09	0.08
**C18:0**	4.45	2.07	3.58	5.55	1.96	1.49	4.32	1.55	1.93	1.62
**C18:1**	6.22	21.53	5.24	5.33	12.96	34.43	26.18	24.08	12.69	8.60
Δ9*c*-18:1 (O)	81.07	89.83	92.42	75.42	82.13	95.49	97.62	96.05	86.00	75.86
Δ11*c*-18:1	17.32	9.83	7.58	21.75	17.20	4.36	2.30	3.86	13.43	23.20
18:1X1	1.61	0.34		2.84	0.68	0.15	0.08	0.09	0.57	0.94
**C18:2**	**45.10**	25.97	**35.73**	**38.93**	29.29	**48.36**	**46.06**	**44.96**	32.53	27.73
Δ9*c*,12*c*-18:2 (L)	99.81	99.76	99.78	99.67	99.78	99.90	99.84	99.87	99.81	99.73
Δ9*c*,12*t*-18:2	0.15	0.24	0.18	0.24	0.22	0.09	0.13	0.13	0.19	0.27
Δ9*t*,12*c*-18:2	0.04		0.04	0.09		0.01	0.03			
**C18:3**	13.49	**28.27**	21.96	13.37	**33.92**	2.24	6.20	8.61	**32.61**	**38.85**
Δ9*c*,12*c*,15*c*-18:3 (Ln)	99.54	99.58	99.48	99.38	99.61	99.56	99.47	99.44	99.64	99.60
*ttc*/*cct*-18:3	0.46	0.42	0.52	0.62	0.39	0.44	0.53	0.56	0.36	0.40
**C19:0**	0.02	0.03	0.02	0.04	0.03	0.01	0.04	0.04	0.02	0.03
**C20:0**	0.50	0.43	0.77	0.57	0.37	0.52	0.47	0.45	0.36	0.31
**C20:1**	0.11	0.15	0.29	0.10	0.12	0.40	0.28	0.60	0.14	0.10
**C20:2**	0.03	0.02	0.09	0.03	0.03	0.05	0.05	0.08	0.03	0.03
**C20:3**	0.00	0.02	0.05	0.00	0.02	0.00	0.00	0.02	0.02	0.02
**C22:0**	0.36	0.82	1.60	0.40	0.51	0.30	0.12	0.45	0.55	0.42
**C22:1**	0.00	0.00	0.02	0.00	0.01	0.01	0.01	0.10	0.00	0.00
**C23:0**	0.16	0.24	0.24	0.24	0.19	0.07	0.06	0.18	0.18	0.25
**C24:0**	0.67	0.54	1.26	0.79	0.58	0.14	0.24	0.36	0.66	0.61
**C24:1**	0.00	0.00	0.00	0.00	0.00	0.00	0.00	0.03	0.00	0.00
**C25:0**	0.06	0.05	0.07	0.08	0.10	0.01	0.04	0.07	0.07	0.07
**C26:0**	0.04	0.03	0.09	0.04	0.08	0.01	0.05	0.03	0.05	0.04
**Sum**	100.00	100.00	100.00	100.00	100.00	100.00	100.00	100.00	100.00	100.00

^a^ Carbon number:degree of unsaturation. ^b^ Abbreviations: *cis*—*c*; *trans*—*t*; palmitoleic—Po; oleic—O; linoleic—L; linolenic—Ln. ^c^ Fatty acids marked with an X are unidentified double bond isomers.

**Table 2:** Mole percentages of squalene, total oxo-fatty acids, and total non-oxo fatty acids in ten pulse samples as determined from GC-FID peak areas.

Analyte	Baby Lima Beans	Black Beans	Black-Eyed Peas	Butter Beans	Cranberry Beans	Garbanzo Beans	Green Split Peas	Lentils	Navy Beans	Pinto Beans
**Squalene ^a^**	0.18	0.22	0.02	0.04	0.16	0.00	0.02	0.00	0.70	0.14
**oxo-FA**	0.33	0.55	1.53	0.09	0.99	0.75	2.52	3.29	0.38	0.29
**Non-oxo-FA**	99.49	99.23	98.45	99.87	98.84	99.24	97.46	96.71	98.92	99.57
**Sum**	100.00	100.00	100.00	100.00	100.00	100.00	100.00	100.00	100.00	100.00

^a^ Squalene was not detected in lentils. It was observed for garbanzo beans but at less than 0.01.

**Table 3:** Distributions of non-oxygenated unsaturated 18-carbon fatty acids and corresponding oxygenated 18-carbon fatty acids relative to their sums as determined from GC-FID peak areas. Isomers are grouped together.

	Baby Lima Beans	Black Beans	Black-Eyed Peas	Butter Beans	Cranberry Beans	Garbanzo Beans	Green Split Peas	Lentils	Navy Beans	Pinto Beans
**18:1**	9.60	28.42	8.33	9.28	17.01	40.51	33.38	31.01	16.31	11.44
**18:2**	69.59	34.28	56.78	67.52	38.46	56.85	58.71	57.89	41.79	36.89
**18:3**	20.81	37.31	34.89	23.19	44.53	2.65	7.90	11.09	41.90	51.67
**Sum**	100.00	100.00	100.00	100.00	100.00	100.00	100.00	100.00	100.00	100.00
**oxo-O**	0.00	0.00	2.92	0.00	0.00	20.63	5.58	24.47	0.00	0.00
**oxo-L**	19.44	2.78	27.55	53.36	1.88	72.88	70.02	52.54	0.00	0.00
**oxo-Ln ^a^**	80.56	97.22	69.53	46.64	98.12	6.49	24.40	22.99	100.00	100.00
**Sum**	100.00	100.00	100.00	100.00	100.00	100.00	100.00	100.00	100.00	100.00

^a^ Includes contribution from suspected oxygenated 18:3 isomer for which signal level was insufficient for MS confirmation.

**Table 4:** Distributions of oxygenated 18-carbon fatty acid isomers expressed as percentages of total oxygenated fatty acid content as determined from GC-FID peak areas.

	Baby Lima Beans	Black Beans	Black-Eyed Peas	Butter Beans	Cranberry Beans	Garbanzo Beans	Green Split Peas	Lentils	Navy Beans	Pinto Beans
**oxo-O**	0.00	0.00	2.92	0.00	0.00	20.63	5.58	24.47	0.00	0.00
**oxo-L1**	13.53	2.78	5.83	28.16	1.88	24.58	61.11	20.90	0.00	0.00
**oxo-L2**	5.91	0.00	21.72	25.20	0.00	48.30	8.91	31.63	0.00	0.00
**oxo-Ln? ^a^**	0.00	0.00	1.41	0.00	1.85	0.82	9.82	6.81	0.00	0.00
**oxo-Ln1**	80.56	97.22	32.03	46.64	96.26	2.99	11.21	2.51	100.00	100.00
**oxo-Ln2**	0.00	0.00	36.09	0.00	0.00	2.68	3.36	13.68	0.00	0.00
**Sum**	100.00	100.00	100.00	100.00	100.00	100.00	100.00	100.00	100.00	100.00

^a^ RT indicates likely oxo-Ln isomer, but identity not confirmed by PCI-CH_4_ MS due to low signal level.

## Data Availability

The data presented in this study are available on request from the corresponding author.

## Sample Availability

The last remaining samples were used for this study. Similar commercially available pulse samples are available.
